# Dendritic cells activate pyroptosis and effector-triggered apoptosis to restrict *Legionella* infection

**DOI:** 10.1128/mbio.01257-25

**Published:** 2025-06-18

**Authors:** Víctor R. Vázquez Marrero, Jessica Doerner, Kimberly A. Wodzanowski, Jenna Zhang, Allyson Lu, Frankie D. Boyer, Isabel Vargas, Suzana Hossain, Karly B. Kammann, Madison V. Dresler, Sunny Shin

**Affiliations:** 1 Department of Microbiology, University of Pennsylvania, Perelman School of Medicine 6572, Philadelphia, Pennsylvania, USA; 2 Department of Biology, University of Pennsylvania, Philadelphia, Pennsylvania, United States; 3 Belmont University 5715, Nashville, Tennessee, USA; 4 Department of Biological Sciences, Mount Holyoke College 7397, South Hadley, Massachusetts, USA; Yale University School of Medicine, New Haven, Connecticut, USA

**Keywords:** *Legionella pneumophila*, dendritic cells, pyroptosis, apoptosis, guard immunity, innate immunity, caspase-11

## Abstract

The innate immune system relies on pattern recognition receptors (PRRs) to detect pathogen-associated molecular patterns (PAMPs) and guard proteins to monitor pathogen disruption of host cell processes. How different immune cell types engage PRRs and guard proteins to respond to infection is poorly understood. Here, we show that macrophages and dendritic cells (DCs) distinctly respond to bacterial virulence activities. In macrophages, the bacterial pathogen *Legionella pneumophila* deploys its Dot/Icm type IV secretion system (T4SS) to deliver effector proteins that facilitate robust intracellular replication. In contrast, T4SS activity triggers rapid death of DCs, which potently restricts *Legionella* replication. Intriguingly, we found that infected DCs exhibit considerable heterogeneity at the single-cell level. Initially, some DCs activate caspase-11 and NLRP3 inflammasome-dependent pyroptosis early during infection. At later time points, other DCs undergo apoptosis driven by T4SS effectors that block host protein synthesis, thereby depleting the pro-survival proteins Mcl-1 and cFLIP. Together, pyroptosis and effector-triggered apoptosis robustly restrict *Legionella* replication in DCs. Collectively, our findings suggest a model where Mcl-1 and cFLIP guard host translation in DCs. Furthermore, our work shows that macrophages and DCs distinctly employ innate immune sensors and guard proteins to mount divergent responses to *Legionella* infection.

**IMPORTANCE:**

The innate immune system senses bacterial pathogens by employing pattern recognition receptors that detect pathogen-associated molecular patterns (PAMPs) and guard proteins that monitor pathogen disruption of host cell processes. How different immune cell types engage pattern recognition receptors (PRRs) and guard proteins to respond to infection is poorly understood. Here, we reveal how dendritic cells (DCs) detect and restrict the intracellular bacterial pathogen *Legionella pneumophila*. At the single-cell level, we find that early during infection, some DCs activate caspase-11 pyroptosis. At later time points, other DCs undergo apoptosis driven by type IV secretion system (T4SS) effectors that block host protein synthesis, which depletes levels of the pro-survival proteins Mcl-1 and cFLIP. Our findings suggest Mcl-1 and cFLIP safeguard mRNA translation in DCs and highlight differences in how macrophages and DCs employ PRRs and guard proteins to respond to bacterial infection.

## INTRODUCTION

Upon infection, host cells utilize pattern recognition receptors (PRRs) to recognize pathogen-associated molecular patterns (PAMPs), resulting in protective immune responses (1). PAMPs are shared by both commensals and pathogens. Thus, to mount appropriate responses to pathogens, hosts additionally sense “patterns of pathogenesis,” such as cytosolic invasion or effector-mediated disruptions (2). PAMPs contaminating the host cell cytosol are detected by cytosolic innate immune sensors, while virulence factor-induced disruptions of host cell processes are sensed by “guard” proteins, a strategy known as “effector-triggered immunity” (ETI) or “guard immunity” (3, 4). Ultimately, detection of patterns of pathogenesis can lead to cell death and elimination of intracellular pathogens. The bacterial virulence activities that induce ETI and how different cell types employ PRRs and guard proteins to sense bacterial infection remain poorly understood. Using opportunistic bacteria like *Legionella pneumophila*, which engages several virulence activities and induces strong protective responses, can help us learn more about these immune mechanisms (5–21).

*Legionella* is naturally found in freshwater environments, where it replicates within amoebae (22–24). Humans are infected with *Legionella* upon inhalation of contaminated water droplets, enabling the bacteria to colonize the lungs and cause the severe pneumonia Legionnaires’ disease (25–27). Although *Legionella* causes significant morbidity and mortality in the elderly and immunocompromised, most healthy individuals successfully clear the infection (28–30). *Legionella* has evolved to target eukaryotic processes highly conserved from amoebae to humans, but has not adapted to evade mammalian immunity (22–24, 31). Thus, *Legionella* serves as an excellent model pathogen for uncovering innate immune mechanisms that may be evaded by mammalian-adapted pathogens.

In the lung, alveolar macrophages are the first immune cell type that encounters *Legionella* and also serve as the primary bacterial replicative niche (27, 32, 33). These cells sense *Legionella* with several cytosolic receptors that initiate assembly of multi-protein complexes termed inflammasomes (34–41). The NAIP5/NLRC4 inflammasome is activated by cytosolic flagellin (35, 38, 39), the NLRP3 inflammasome is activated by type IV secretion system (T4SS) activity (34, 36, 37), and the caspase-11 inflammasome is activated by cytosolic lipopolysaccharide (LPS) (36, 37, 40, 41). These inflammasomes lead to the activation of caspases-1 and -11, which cleave the pore-forming protein gasdermin D (GSDMD) and IL-1 family cytokines into their active forms (42–44). These events cause an inflammatory cell death known as pyroptosis, which eliminates the infected cell and alerts nearby cells to help control infection (45, 46). NAIP5/NLRC4-dependent pyroptosis restricts *Legionella* replication within unprimed macrophages (35, 38, 39, 47, 48), whereas the NLRP3 and caspase-11 inflammasomes do not and instead promote IL-1 cytokine release (36, 37, 40).

*Legionella-*infected macrophages also activate ETI upon sensing bacterial perturbation of host processes. *Legionella* injects over 300 effectors via its Dot/Icm T4SS to establish an endoplasmic reticulum-derived vacuole that supports bacterial replication (8, 33, 49–51). These effectors manipulate multiple eukaryotic processes, including membrane trafficking and mRNA translation (31, 52). At least seven *Legionella* effectors (Lgt1-3, SidI, SidL, LegK4, and RavX) block mRNA translation, leading to a >95% decrease in *de novo* protein synthesis (12, 15, 18, 19, 53–56). Proteins that undergo rapid turnover, like the nuclear factor-kappa B (NF-κB) negative regulators IκB and A20, are particularly sensitive to *Legionella* disrupting mRNA translation, leading to sustained NF-κB signaling and upregulation of inflammatory genes (18). Thus, IκB and A20 serve as guards of host protein synthesis. Similarly, pro-survival proteins from the B-cell lymphoma-2 (Bcl-2) family guard mRNA translation during viral infection of human keratinocytes (57). Simultaneous downregulation of the short-lived protein myeloid cell leukemia-1 (Mcl-1) and inactivation of Bcl-XL leads to caspase-3-dependent cleavage of gasdermin E (GSDME), which triggers pyroptosis to restrict viral replication (57). Whether these pro-survival proteins sense translational shutdown in other cell types or during bacterial infection is unclear.

Many studies focus on *Legionella* interactions with macrophages, their primary replicative niche during pulmonary infection (27, 32, 33), but other lung immune cells also interact with and respond to *Legionella*. For example, neutrophils take up *Legionella* (32), and both neutrophils and monocytes promote pro-inflammatory responses and control of infection (45, 46, 58–61). Later during infection, a robust T and B cell response is generated (62–64). However, the interaction between *Legionella* and dendritic cells (DCs) is less well understood. In other contexts, DCs bridge innate and adaptive immune responses by acting as antigen-presenting cells (65–67). Unlike macrophages, DCs are not productively infected during *in vivo Legionella* infection (32). *In vitro*, DCs undergo rapid caspase-3-dependent apoptosis upon detecting *Legionella* T4SS activity, thereby restricting bacterial replication (68, 69). These findings suggest that, unlike macrophages, DCs possess unique mechanisms that promote apoptosis and restrict bacterial replication in response to T4SS-translocated products.

Here, we investigated the host pathways and bacterial effectors that mediate cell death and restriction of *Legionella* replication in DCs. We found that apoptosis was triggered by *Legionella* T4SS effectors that block host protein synthesis. DCs express substantially lower levels of the pro-survival proteins Mcl-1 and cellular FADD-like IL-1β-converting enzyme-inhibitory protein (cFLIP) than macrophages, and effector blockade of host translation led to decreased levels of these proteins. We also found considerable heterogeneity at the single-cell level, as DCs activated *either* caspase-11 and NLRP3 inflammasome-dependent pyroptosis early during infection *or* apoptosis later during infection. Both apoptosis and pyroptosis promoted restriction of intracellular *Legionella* replication. Altogether, we show that DCs activate either PRR-triggered pyroptosis or effector-triggered apoptosis to restrict *Legionella* replication. Our findings suggest a model where Mcl-1 and cFLIP guard host protein synthesis in DCs, and pathogen interference with host translation decreases levels of these proteins, thus triggering apoptosis. Furthermore, our findings also highlight major differences in the PRRs and guard proteins used by DCs and macrophages to mount distinct immune responses to *Legionella* infection.

## RESULTS

### Dendritic cells trigger both extrinsic and intrinsic apoptosis in response to *Legionella pneumophila* infection

**Fig 1:**
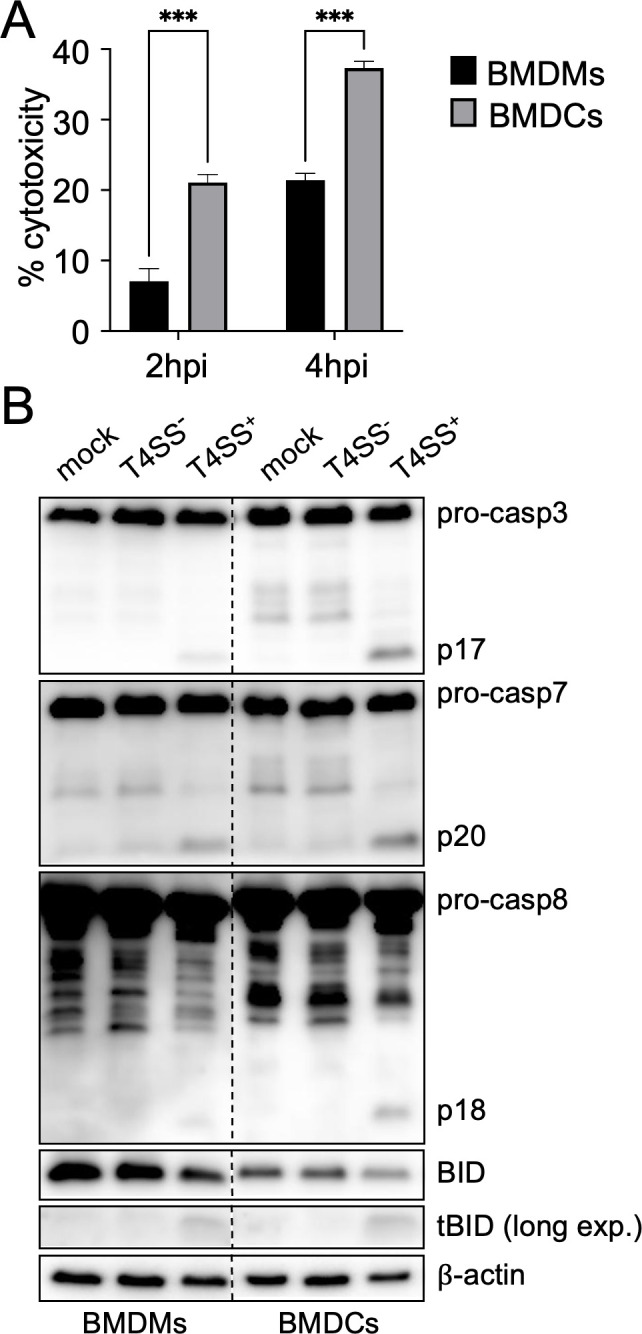
Dendritic cells undergo both cell-extrinsic and intrinsic apoptosis in response to *Legionella* infection. (**A**) BMDMs and BMDCs were infected with non-replicating *thyA*Δ*flaA Legionella* (T4SS^+^) at a multiplicity of infection (MOI) of 50 for 2 or 4 h. Cytotoxicity was measured by LDH release assay. (**B**) BMDMs and BMDCs were mock-infected or infected with *thyA*Δ*flaA*Δ*dotA* (T4SS^−^) or T4SS^+^ *Legionella* at an MOI of 50 for 4 h. Immunoblot analysis was performed on cell lysates for caspase-3, caspase-7, caspase-8, BID, and β-actin as a loading control. Shown are lanes that were cropped from the same immunoblots, with the dashed line denoting where lanes were cropped. long exp., long exposure; hpi, hours post-infection. Data shown are representative of at least two (**B**) or three (**A**) independent experiments. Graphs show the mean ± SEM of triplicate wells. Data were analyzed by two-way analysis of variance (ANOVA) with Šídák’s post-test; ***, *P* < 0.001.

In contrast to murine bone marrow-derived macrophages (BMDMs) differentiated with macrophage-colony stimulating factor (M-CSF), bone marrow-derived dendritic cells (BMDCs) differentiated with granulocyte macrophage-colony stimulating factor (GM-CSF) are not permissive for intracellular *Legionella* replication as they undergo rapid mitochondrial apoptosis during infection (68, 69). We sought to investigate the bacterial and host factors that mediate this response. To avoid flagellin-mediated activation of the NAIP5/NLRC4 inflammasome, which restricts bacterial replication in both macrophages and DCs and can potentially mask other cell death mechanisms, we employed flagellin-deficient Δ*flaA Legionella* (hereafter T4SS^+^) (38, 39, 50, 69). To circumvent any confounding effects of intracellular bacterial replication, we also used thymidine auxotrophic *Legionella* strains incapable of replicating in the absence of exogenous thymidine. We first assessed cell death kinetics in infected BMDMs and BMDCs by monitoring the extracellular release of lactate dehydrogenase (LDH). As expected, cell death levels were significantly higher in BMDCs at 2 and 4 h post-infection compared to BMDMs, indicating that DCs undergo more robust and rapid cell death than macrophages in response to *Legionella* (Fig. 1A).

We next investigated the apoptotic pathways induced by *Legionella* in DCs. Previous studies found that pro-apoptotic proteins Bcl2-associated X protein (BAX) and Bcl2 antagonist/killer protein (BAK) activate caspase-3 and restrict *Legionella* replication in BMDCs via the intrinsic mitochondrial apoptosis pathway (69). However, caspase-3 can also be activated by caspase-8 during extrinsic apoptosis, with crosstalk occurring through caspase-8-mediated cleavage of BH3-interacting domain death agonist (BID) into its truncated form (tBID), which activates BAX and BAK (70). Indeed, DCs infected with T4SS^+^ *Legionella* showed increased cleavage of caspase-8 and BID, as well as caspases-3 and -7, compared to BMDMs (Fig. 1B). Caspase cleavage relied on a functional T4SS, as DCs infected with Δ*flaA*Δ*dotA Legionella* lacking the essential T4SS component DotA (hereafter T4SS^−^) did not exhibit cleavage of caspases-3, -7, -8, or BID (Fig. 1B). To address the role of caspase-8 in caspase-3 and -7 cleavage, we infected BMDCs lacking both caspase-8 and receptor-interacting protein kinase 3 (RIPK3), since caspase-8-deficient mice exhibit uncontrolled activation of RIPK3-mediated necroptosis and are embryonically lethal (71–73). Immunoblot analysis revealed partially decreased caspase-3 and -7 cleavage in *Ripk3^−/−^Casp8^−/−^* BMDCs compared to WT or *Ripk3^−/−^* BMDCs following T4SS^+^ *Legionella* infection (Fig. S1), indicating that caspase-8 is not the sole driver of DC apoptosis and that intrinsic apoptosis is also activated, as previously reported (69). Altogether, these results show that DCs undergo both extrinsic and intrinsic apoptosis in response to *Legionella* T4SS activity.

### *Legionella* T4SS effectors that block host translation trigger apoptosis in dendritic cells

We next aimed to determine the T4SS-dependent bacterial factors responsible for inducing apoptosis in *Legionella-*infected BMDCs. At homeostasis, apoptosis is inhibited by the pro-survival Bcl-2 family of proteins (74, 75). Some Bcl-2 family members, like Mcl-1, have a short half-life of 0.5 h (76, 77). Thus, inhibition of host protein synthesis by antibiotics like cycloheximide or microbial toxins leads to rapid depletion of Mcl-1 and other pro-survival proteins, causing some cell types to undergo apoptosis (57, 78–81). Since *Legionella* has at least seven effectors that potently and redundantly block mRNA translation (12, 15, 18, 19, 53–56), we hypothesized these effectors trigger apoptosis in DCs.

**Fig 2:**
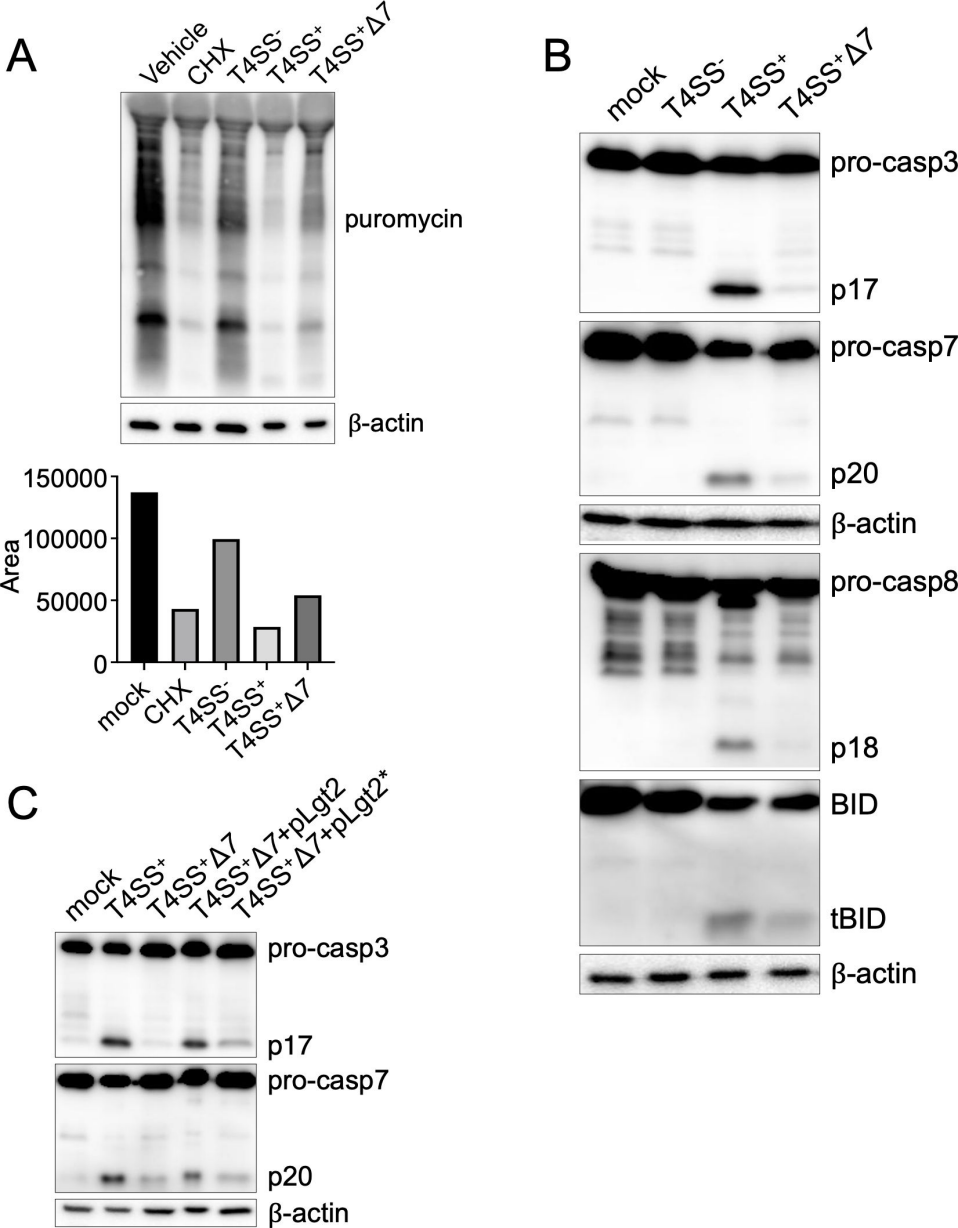
A subset of *Legionella* T4SS effectors block host protein synthesis and trigger apoptosis in dendritic cells. (**A**) BMDCs were infected with T4SS^+^ *Legionella* at an MOI of 10 or treated with cycloheximide for 4 h. A 10 µg/mL puromycin was added for 1 h, and cell lysates were harvested. Immunoblot analysis with anti-puromycin antibody was performed on cell lysates. β-actin was used as a loading control. Band density was quantified using ImageJ; the bar graph shows the corresponding area under the curve. (**B**) BMDCs were mock-infected or infected with T4SS^−^, T4SS^+^, or *thyA*Δ*flaA*Δ*7 Legionella* (T4SS^+^Δ7) at an MOI of 50 for 4 h. Immunoblot analysis was performed on cell lysates for caspase-3, caspase-7, caspase-8, BID, and β-actin as a loading control. (**C**) BMDCs were mock-infected or infected with T4SS^+^, T4SS^+^Δ*7*, T4SS^+^Δ*7*+pLgt2, and T4SS^+^Δ*7*+pLgt2* *Legionella*. Lysates were analyzed by immunoblot for caspase-3, caspase-7, and β-actin as a loading control. The data shown are representative of at least three independent experiments.

While *Legionella* inhibits mRNA translation in macrophages (18), whether it also does so in DCs is unknown. We therefore monitored global protein synthesis in BMDCs with surface sensing of translation (SUnSET) (82), which uses minimal amounts of the aminoacyl tRNA analog puromycin together with anti-puromycin antibodies to detect puromycin incorporation into nascently translated peptides. Immunoblot-based quantification of puromycin incorporation showed that BMDCs infected with T4SS^+^ *Legionella* had reduced levels of protein synthesis similar to those seen in cycloheximide-treated BMDCs, compared to vehicle-treated cells (Fig. 2A). In contrast, T4SS^−^ *Legionella-*infected cells exhibited robust protein synthesis. DCs infected with *Legionella* lacking seven effectors that block host translation (hereafter T4SS^+^Δ*7*) (55) displayed substantially higher levels of protein synthesis compared to T4SS^+^ *Legionella-*infected DCs (Fig. 2A), indicating that these effectors inhibit translation in DCs. Protein synthesis levels in T4SS^+^Δ*7 Legionella*-infected DCs were not fully restored to the levels in T4SS^−^ *Legionella*-infected DCs, in agreement with studies indicating that T4SS^+^Δ*7 Legionella* still partially blocks host protein synthesis (55), suggesting that *Legionella* has additional effectors that block host translation.

We next examined whether the *Legionella* effectors that block host protein synthesis induce apoptosis in DCs. DCs infected with T4SS^+^Δ*7 Legionella* had reduced cleavage of caspases-8, -3, -7, and BID compared to T4SS^+^ *Legionella-*infected cells (Fig. 2B). Caspase-3 and -7 cleavage were restored by genetically complementing T4SS^+^Δ*7 Legionella* with one of the seven effectors, *Legionella* glucosyltransferase 2 (Lgt2) (Fig. 2C). In contrast, complementing T4SS^+^Δ*7 Legionella* with a catalytically inactive Lgt2 mutant unable to block host protein synthesis (18) did not restore caspase cleavage, indicating that Lgt2’s catalytic activity is required for DCs to activate apoptosis. Altogether, these data show that a subset of *Legionella* T4SS effectors robustly block host protein synthesis and trigger apoptosis in infected DCs.

### Dendritic cells express low levels of anti-apoptotic proteins that are further reduced due to *Legionella*’s blockade of host protein synthesis

**Fig 3:**
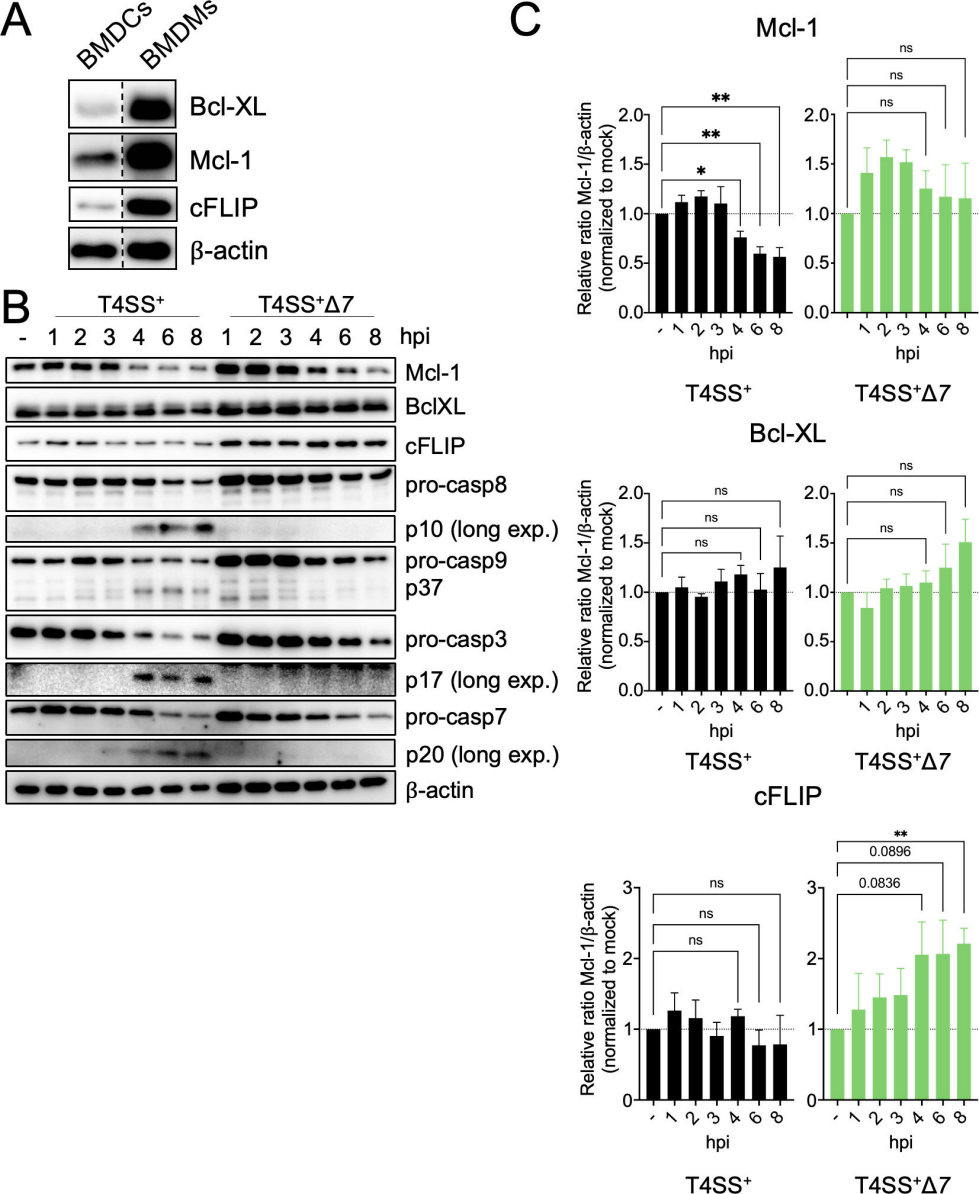
Dendritic cells express low levels of Mcl-1 and cFLIP that are further decreased by *Legionella* T4SS effectors. (**A**) Immunoblot analysis was performed on uninfected BMDM and BMDC lysates for Bcl-XL, Mcl-1, cFLIP, and β-actin as a loading control. Shown are lanes that were cropped from the same immunoblots, with the dashed line denoting where lanes were cropped. (**B**) BMDCs were mock-infected (represented as “−“) or infected with T4SS^+^ or T4SS^+^Δ*7 Legionella* at an MOI of 50 for 1, 2, 3, 4, 6, or 8 h. Immunoblot analysis was performed on cell lysates for Mcl-1, Bcl-XL, cFLIP, caspase-8, caspase-9, caspase-3, caspase-7, and β-actin as a loading control. The immunoblot was subjected to a longer exposure time to visualize the cleaved forms of caspases-8, -3, and -7. (**C**) Three independent experiments from panel B were quantified using ImageJ for Bcl-XL, Mcl-1, and cFLIP, and the average was graphed for each protein. Graphs show the mean ± SEM of triplicate wells. Data shown are representative of at least (**A**) two and (**B**) three independent experiments. Data in panel C were analyzed by unpaired Student’s *t*-test; **, *P* < 0.01; *, *P* < 0.05; ns, not significant.

Intrinsic apoptosis is regulated by the balance between pro- and anti-apoptotic proteins, many belonging to the Bcl-2 family of proteins (74, 75). Several Bcl-2 family members, including Mcl-1, have a relatively short half-life, and depletion of these pro-survival factors due to chemical inhibitors or microbial toxins leads to apoptosis in other cells (57, 78–81). Notably, Mcl-1 and Bcl-XL have been described as guards of host translation during viral infection of keratinocytes (57). Extrinsic apoptosis is regulated by an anti-apoptotic caspase-8 homolog called cFLIP. cFLIP is upregulated by NF-κB-activating stimuli, promoting formation of the caspase-8:cFLIP heterodimer, which blocks apoptosis and promotes cellular survival (83, 84). When cFLIP fails to be upregulated due to chemical or bacterial blockade of NF-κB signaling or host translation (85–88), caspase-8 instead forms a homodimer that drives apoptosis (89, 90). Since DCs undergo both intrinsic and extrinsic apoptosis in response to *Legionella* blockade of host translation, whereas macrophages do not, we examined whether DCs basally express lower levels of pro-survival factors than macrophages. Notably, DCs express lower levels of the pro-survival proteins Bcl-XL, Mcl-1, and cFLIP than macrophages (Fig. 3A). Thus, lower expression of pro-survival factors in DCs may render them more susceptible to apoptosis than macrophages upon bacterial inhibition of protein synthesis.

We next determined whether pro-survival protein levels are reduced by *Legionella* effector-mediated blockade of host translation. T4SS^+^ *Legionella*-infected DCs had significantly decreased Mcl-1 levels by 4 h post-infection compared to uninfected cells (Fig. 3B and C). In contrast, in T4SS^+^Δ*7 Legionella*-infected DCs*,* Mcl-1 levels were slightly decreased but remained higher than Mcl-1 levels in T4SS^+^ *Legionella*-infected DCs (Fig. 3B and C). Bcl-XL levels were unaffected by the seven *Legionella* effectors (Fig. 3B and C), possibly due to its long half-life of 20 h (91). cFLIP levels in uninfected and T4SS^+^ *Legionella*-infected DCs were similar, whereas cFLIP levels were significantly upregulated in T4SS^+^Δ*7 Legionella*-infected cells, suggesting that the seven *Legionella* effectors prevent cFLIP upregulation. In T4SS^+^ *Legionella-*infected DCs, cleavage of caspases-8, -9, -3, and -7 began at 4 h post-infection, coinciding with the decrease in Mcl-1 levels (Fig. 3B and C). In contrast, there was no apoptotic caspase cleavage in T4SS^+^Δ*7 Legionella*-infected DCs. Taken together, these data indicate that in contrast to macrophages, DCs express lower levels of the critical pro-survival factors Mcl-1 and cFLIP that regulate intrinsic and extrinsic apoptosis, respectively. This finding potentially explains why DCs undergo rapid apoptosis in response to *Legionella,* whereas macrophages do not. Additionally, these results suggest a model where pro-survival factors are rapidly depleted or fail to be upregulated in DCs due to the *Legionella* effector-mediated block of host translation, thereby directing DCs to die by apoptosis.

### Dendritic cells undergo caspase-1-, caspase-11-, and gasdermin D-dependent pyroptosis early during *Legionella* infection

Our analysis revealed that the *Legionella* effectors that block host protein synthesis trigger apoptotic caspase cleavage at around 4 h post-infection (Fig. 3B). However, cell death occurs as early as 2 h post-infection (Fig. 1A). To more rigorously assess cell death kinetics, we measured propidium iodide (PI) uptake over time in DCs infected with T4SS^+^ or T4SS^+^Δ*7 Legionella*. We observed only a partial decrease in cell death in T4SS^+^Δ*7 Legionella*-infected DCs compared to T4SS^+^ *Legionella*-infected DCs (Fig. S2), despite the absence of apoptotic caspase cleavage in these cells (Fig. 3B). These data indicate that effector-triggered apoptosis does not account for all of the cell death occurring in infected DCs.

**Fig 4:**
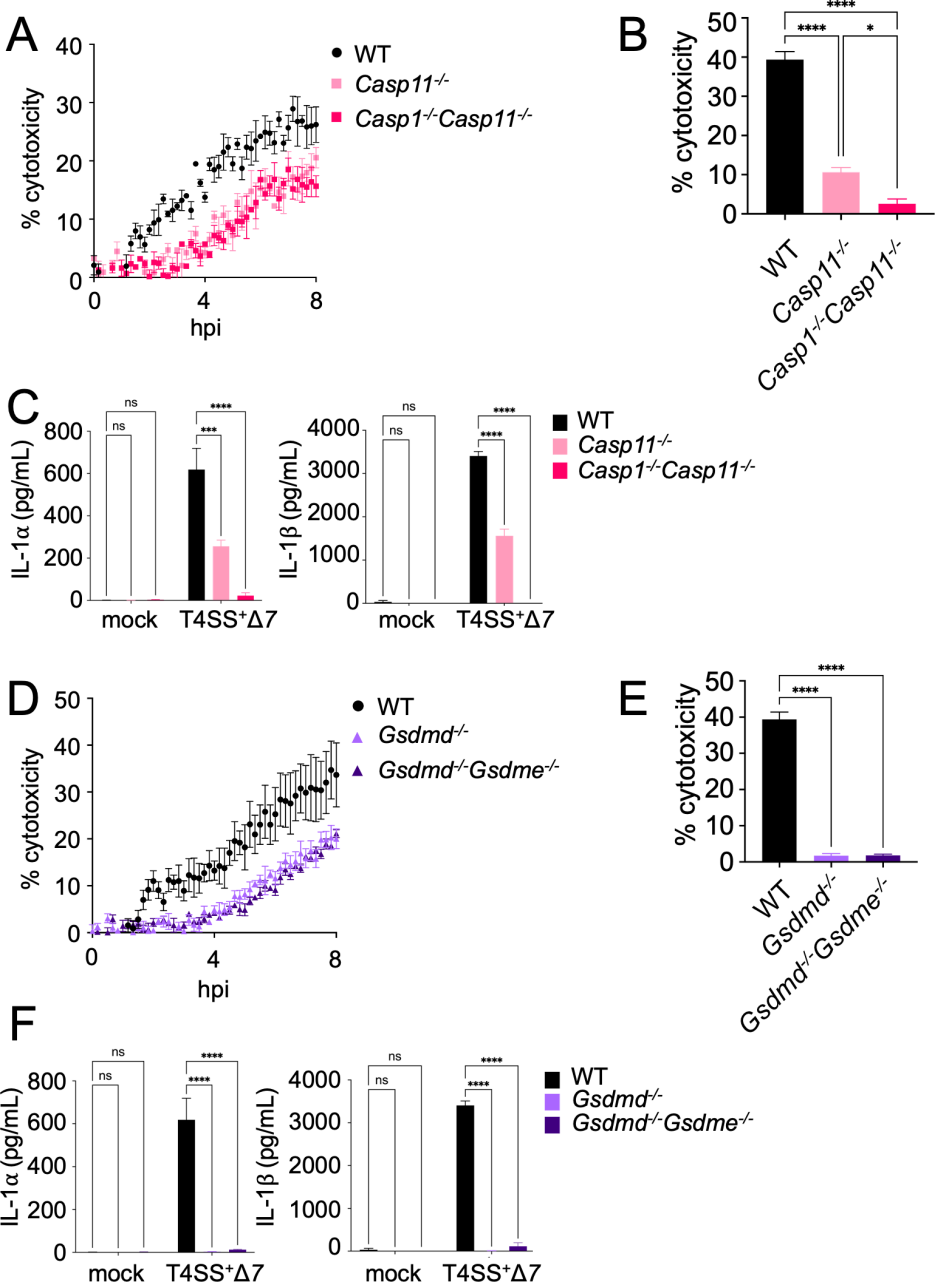
Dendritic cells undergo rapid pyroptosis in response to *Legionella* infection. (**A through C**) WT, *Casp11^−/−^*, or *Casp1^−/−^Casp11^−/−^* BMDCs were mock-infected (**C**) or infected with T4SS^+^ (**A, B**) or T4SS^+^Δ*7 Legionella* (**C**) at an MOI of 50. Cytotoxicity was measured by PI uptake assay (**A**) or LDH release assay at 4 h (**B**). Cytokine release was measured at 4 h by enzyme-linked immunosorbent assay (ELISA) (**C**). (**D through F**) WT, *Gsdmd^−/−^*, or *Gsdmd^−/−^Gsdme^−/−^* BMDCs were mock-infected (**F**) or infected with T4SS^+^ (**D, E**) or T4SS^+^Δ*7 Legionella* (**F**) at an MOI of 50. Cytotoxicity was measured by PI uptake (**D**) or LDH release assay at 4 h (**E**). Cytokine release was measured at 4 h by ELISA (**F**). Data shown are representative of at least three independent experiments. Graphs show the mean ± SEM of triplicate wells. Data were analyzed by one-way ANOVA with Tukey honestly significant difference (HSD) post-test (**B, E**) or two-way ANOVA with Dunnett’s post-test (**C, F**); ****, *P* < 0.0001; ***, *P* < 0.001; *, *P* < 0.05; ns, not significant.

We next investigated what additional forms of cell death occur in *Legionella-*infected DCs. In macrophages, *Legionella* activates the NAIP5/NLRC4 and caspase-11 inflammasomes (35–41), which lead to pyroptosis (92). In DCs, *Legionella* also activate the NAIP5/NLRC4 inflammasome (69), but whether additional inflammasomes are activated is unknown. In this study, we use flagellin-deficient *Legionella,* thus eliminating the contribution of the NAIP5/NLRC4 inflammasome. Therefore, we tested whether flagellin-deficient *Legionella* trigger caspase-1 and caspase-11-dependent pyroptosis by infecting WT, *Casp11^−/−^,* or *Casp1^−/−^Casp11^−/−^* BMDCs. Cell death within the first 4 h of infection mainly depended on caspase-11, as measured by PI uptake and LDH release (Fig. 4A and B), with a modest additional decrease in cell death in *Casp1^−/−^Casp11^−/−^* BMDCs compared to *Casp11^−/−^* BMDCs. DCs infected with T4SS^+^ *Legionella* had low levels of IL-1 production due to the block in host protein synthesis (Fig. S3) (5, 93, 94). Therefore, we infected BMDCs with T4SS^+^Δ*7 Legionella* to assess the roles of caspases-1 and -11 in IL-1α and IL-1β release. Deletion of caspase-11 alone resulted in a significant decrease in IL-1α and IL-1β release relative to WT BMDCs, while deletion of both caspases-1 and -11 fully abrogated IL-1 release (Fig. 4C). These data indicate that both caspase-1 and -11 contribute to IL-1 release by *Legionella-*infected DCs.

Caspase-1 and caspase-11 cleave GSDMD, liberating its active pore-forming N-terminal domain, which then oligomerizes in the plasma membrane to form a pyroptotic pore that releases IL-1 family cytokines and damage-associated molecular patterns (42–44, 95, 96). To assess the role of GSDMD in DC pyroptosis, we infected *Gsdmd*^−/−^ BMDCs with T4SS^+^ *Legionella*. We observed substantially less cell death, as measured by PI uptake and LDH release, in *Gsdmd*^−/−^ BMDCs within the first 4 h of infection compared to WT BMDCs (Fig. 4D and E). However, there were still substantial levels of cell death remaining in infected *Gsdmd^−/−^* BMDCs (Fig. 4D). Given that in other circumstances, GSDME can compensate for GSDMD deficiency and mediate pyroptosis (40, 97, 98), we additionally infected *Gsdmd^−/−^Gsdme*^−/−^ BMDCs. However, there was no further reduction in cell death (Fig. 4D and E). IL-1 cytokine release from infected *Gsdmd^−/−^* and *Gsdmd^−/−^Gsdme*^−/−^ BMDCs was nearly completely abrogated (Fig. 4F), indicating that GSDMD, not GSDME, primarily drives pyroptosis in *Legionella*-infected DCs. Collectively, these data indicate that in addition to T4SS effector-triggered apoptosis, DCs activate caspase-11- and GSDMD-dependent pyroptosis early during *Legionella* infection.

Tumor necrosis factor (TNF) signaling can promote both caspase-11-dependent pyroptosis and caspase-8-mediated apoptosis (40, 90). We therefore determined the role of TNF in DC death during *Legionella* infection. Notably, cell death levels were significantly reduced in *Tnf^−/−^* BMDCs infected with T4SS^+^*Legionella* compared to infected WT BMDCs (Fig. S4A and B). We also observed delayed GSDMD processing into its active p32 fragment in infected *Tnf^−/−^* BMDCs but found no defect in apoptotic caspase cleavage (Fig. S4C and D), indicating that TNF facilitates pyroptosis but not apoptosis in *Legionella*-infected DCs. We previously showed that TNF licenses macrophages to upregulate and rapidly activate caspase-11 in response to *Legionella* infection (40). However, caspase-11 protein levels were similar in WT and *Tnf^−/−^* BMDCs infected with T4SS^+^ *Legionella* (Fig. S4C), suggesting that TNF promotes pyroptosis in DCs through other mechanisms.

### Dendritic cells activate the NLRP3 inflammasome to mediate IL-1β release during *Legionella* infection

**Fig 5:**
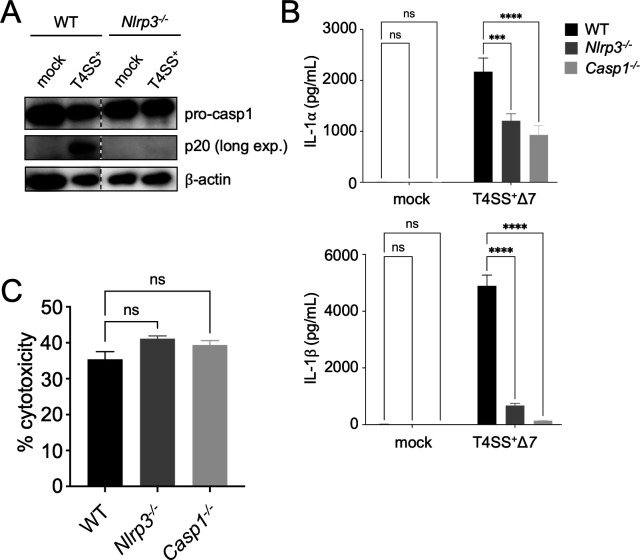
Infected dendritic cells activate the NLRP3 inflammasome to promote caspase-1 activation and IL-1 release. (**A**) WT and *Nlrp3^−/−^* BMDCs were mock-infected or infected with T4SS^+^ *Legionella* at an MOI of 50 for 4 h. Immunoblot analysis was performed on cell lysates for caspase-1 and β-actin as a loading control. The immunoblot was subjected to a longer exposure time to visualize caspase-1 p20. Shown are lanes that were cropped from the same immunoblots, with the dashed line denoting where lanes were cropped. (**B and C**) WT, *Nlrp3^−/−^*, and *Casp1^−/−^* BMDCs were mock-infected (**B**) or infected with T4SS^+^Δ*7* (**B**) or T4SS^+^ (**C**) *Legionella* at an MOI of 50 for 4 h. Cytokine release was measured by ELISA (**B**). Cytotoxicity was measured by LDH release assay (**C**). Data shown are representative of at least three independent experiments. Graphs show the mean ± SEM of triplicate wells. Data were analyzed by one-way ANOVA with Tukey HSD post-test (**C**) or two-way ANOVA with Dunnett’s post-test (**B**); ****, *P* < 0.0001; ***, *P* < 0.001; ns, not significant.

Our data indicate that although caspase-1 is not required for cell death, it is required for IL-1β release from *Legionella-*infected DCs (Fig. 4C). We therefore investigated the pathways activating caspase-1. Caspase-11-dependent GSDMD pore formation in macrophages leads to potassium efflux, which triggers the non-canonical NLRP3 inflammasome and caspase-1 activation to enable IL-1β cleavage and release (36, 37, 99–101). Given that *Legionella* activates both the canonical and non-canonical NLRP3 inflammasomes in mouse macrophages (34, 36, 37, 40) and our data indicate that both caspases-1 and -11 regulate IL-1β release from *Legionella-*infected DCs, we hypothesized that the NLRP3 inflammasome mediates caspase-1-dependent IL-1 release downstream of caspase-11 in infected DCs. Caspase-1 cleavage was substantially decreased in T4SS^+^ *Legionella*-infected *Nlrp3^−/−^* BMDCs, indicating that the NLRP3 inflammasome is required for caspase-1 activation (Fig. 5A). Furthermore, we observed nearly complete abrogation of IL-1β release from T4SS^+^Δ*7 Legionella*-infected *Nlrp3^−/−^* or *Casp1^−/−^* BMDCs compared to WT BMDCs, indicating that the NLRP3 inflammasome is the primary driver of IL-1β release (Fig. 5B). However, there was only a partial decrease in IL-1α release from *Nlrp3^−/−^* or *Casp1^−/−^* BMDCs, whereas IL-1α release was substantially decreased in *Casp1^−/−^Casp11^−/−^* BMDCs (Fig. 4C), indicating that both caspases contribute to IL-1α release. *Nlrp3^−/−^* or *Casp1^−/−^* BMDCs infected with T4SS^+^ *Legionella* exhibited WT levels of cell death (Fig. 5C), indicating that while NLRP3 and caspase-1 drive IL-1β release, they are not required for DC death during *Legionella* infection.

### Dendritic cells activate either pyroptosis or effector-triggered apoptosis to restrict *Legionella* infection

**Fig 6:**
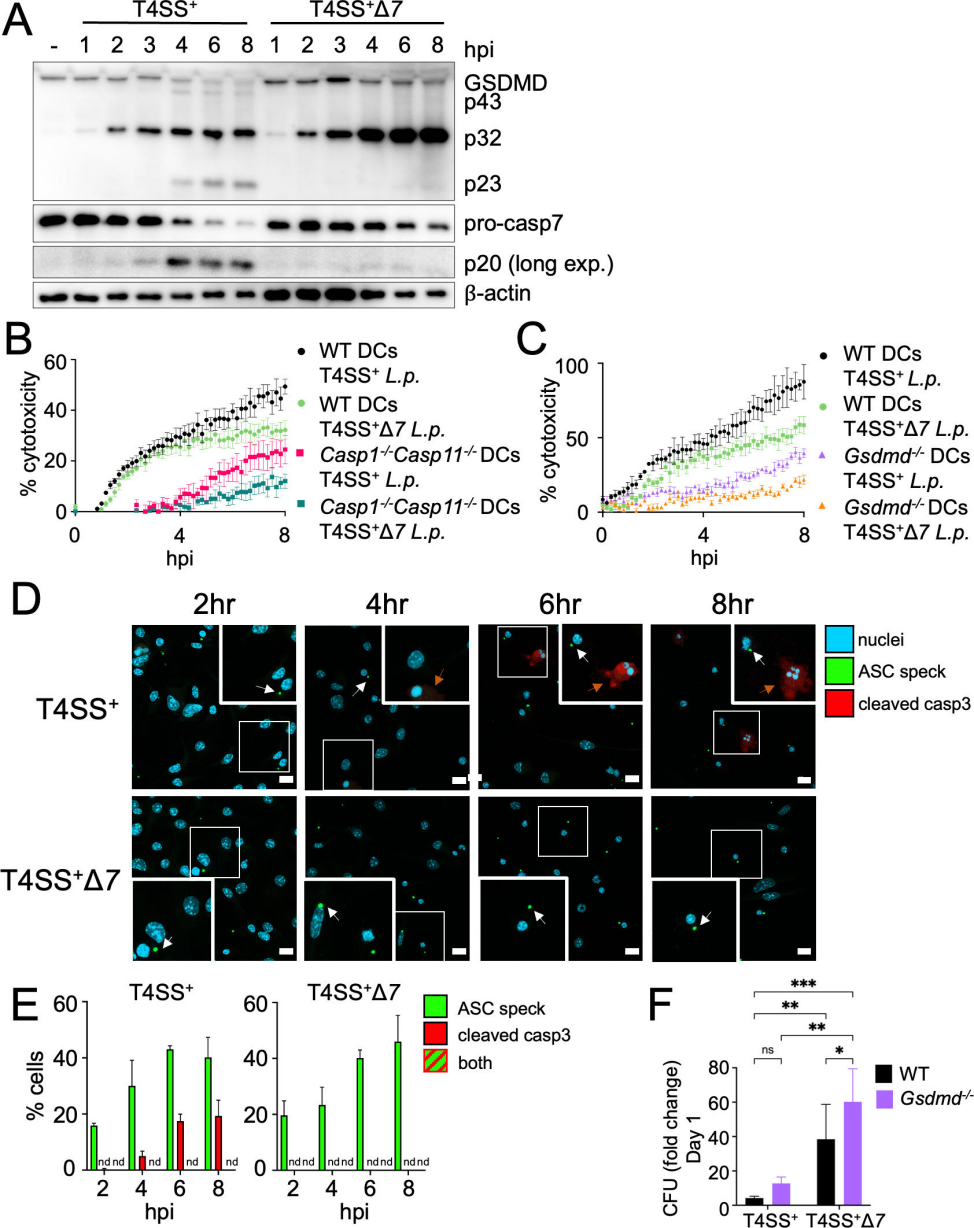
Dendritic cells activate either pyroptosis or effector-triggered apoptosis to restrict *Legionella* infection. (**A**) BMDCs were mock-infected (represented as “−“) or infected with T4SS^+^ or T4SS^+^Δ*7 Legionella* at an MOI of 50 for 1, 2, 3, 4, 6, or 8 h. Immunoblot analysis was performed on cell lysates for GSDMD, caspase-7, and β-actin as a loading control. (**B**) WT or *Casp1^−/−^Casp11^−/−^* BMDCs were infected with T4SS^+^ or T4SS^+^Δ*7 Legionella* at an MOI of 50. Cytotoxicity was measured by PI uptake assay. (**C**) WT or *Gsdmd^−/−^* BMDCs were infected with T4SS^+^ or T4SS^+^Δ*7 Legionella* at an MOI of 50. Cytotoxicity was measured by PI uptake assay. (**D**) ASC-citrine reporter BMDCs were infected with T4SS^+^ or T4SS^+^Δ*7 Legionella* at an MOI of 50 for 2, 4, 6, or 8 h and prepared for confocal microscopy. Cells were stained for cleaved caspase-3 (red) and nuclei (blue) with Hoechst. White arrows point to ASC speck-positive cells, and orange arrows point to cleaved caspase-3-positive cells. Insets represent a 1.75× zoom. Scale bars = 10 µm. (**E**) Cells positive for ASC specks or cleaved caspase-3 from (**D**) were quantified by counting at least 100 total cells per condition for each independent experiment. (**F**) WT and *Gsdmd^−/−^* BMDCs were infected with T4SS^+^ or T4SS^+^Δ*7 Legionella* at an MOI of 10. The fold change in colony-forming units (CFU) was quantified at 1 day post-infection. nd, not detected. (**A, B, C, E**) Data shown are representative of at least three independent experiments. (**D, F**) Data represent the pooled results of at least three independent experiments. Graphs show the mean ± SEM of triplicate wells. Data were analyzed by two-way ANOVA with Šídák’s post-test; **, *P* < 0.01; *, *P* < 0.05; ns, not significant.

*Legionella-*infected DCs activate both pyroptosis and effector-triggered apoptosis, yet the relative kinetics of these pathways remained unclear. We therefore assessed the relative timing of pyroptosis and apoptosis in WT DCs infected with T4SS^+^ or T4SS^+^Δ*7 Legionella*. Pyroptosis was rapidly induced within 1 h of T4SS^+^ or T4SS^+^Δ*7 Legionella* infection, as evidenced by GSDMD processing into its active p32 fragment (Fig. 6A). Later, at 3 to 4 h post-infection, caspase-7 was cleaved into its p20 form following infection with T4SS^+^ *Legionella* but not T4SS^+^Δ*7 Legionella*, indicating that apoptosis is triggered following pyroptosis during T4SS^+^ *Legionella* infection. At 4 h following T4SS^+^ *Legionella* infection, GSDMD was processed into its inactive p23 and p43 fragments, possibly due to the activity of caspases-3 and -7 (102, 103). In contrast, we observed more GSDMD p32 and the absence of p23 and p43 fragments in DCs infected with T4SS^+^Δ*7 Legionella,* suggesting that caspases-3 and -7 limit the formation of active GSDMD p32 during T4SS^+^ *Legionella* infection.

As a complementary approach, we further assessed the kinetics of pyroptosis and apoptosis in infected DCs by measuring PI uptake in WT or *Casp1/11^−/−^* DCs infected with either T4SS^+^ or T4SS^+^Δ*7 Legionella. Casp1/11^−/−^* DCs did not undergo cell death until 4 h following T4SS^+^ *Legionella* infection, whereas WT DCs exhibited rapid cell death within 1 h post-infection (Fig. 6B). WT BMDCs infected with T4SS^+^Δ*7 Legionella* also underwent rapid cell death similar to T4SS^+^ *Legionella*-infected WT DCs within the first 4 h of infection, but had reduced cell death at later time points. In contrast, *Casp1/11^−/−^* DCs infected with T4SS^+^Δ*7 Legionella* showed minimal cell death at all time points examined.

To further define the kinetics of GSDMD-dependent pyroptosis and effector-triggered apoptosis, we next infected WT or *Gsdmd^−/−^* DCs with T4SS^+^ or T4SS^+^Δ*7 Legionella. Gsdmd^−/−^* DCs infected with T4SS^+^ *Legionella* underwent very little cell death at early time points and then exhibited some cell death after 4 h of infection compared to T4SS^+^ *Legionella-*infected WT DCs (Fig. 6C). Consistent with our previous observations in *Casp1/11^−/−^* DCs, cell death was nearly abrogated in T4SS^+^Δ*7 Legionella*-infected *Gsdmd^−/−^* DCs compared to the other conditions. Altogether, these data indicate that infected DCs activate pyroptosis first, followed by effector-triggered apoptosis, and that both pathways account for most of DC death during infection.

Given that the kinetics of pyroptosis and apoptosis partially overlap in T4SS^+^ *Legionella-*infected DCs, we considered whether individual DCs simultaneously experience both pyroptosis and apoptosis or undergo either pyroptosis or apoptosis during infection. During pyroptosis, NLRP3, ASC, and caspase-1 form an inflammasome complex that can be visualized as a large perinuclear speck by microscopy (104–106). We therefore imaged ASC specks using BMDCs expressing an ASC-citrine fusion protein, whereas apoptotic cells were identified by antibody-based staining for cleaved caspase-3. Confocal microscopy analysis revealed that DCs infected with T4SS^+^ or T4SS^+^Δ*7 Legionella* contained ASC specks starting at 2 h post-infection, and the percentage of cells positive for ASC specks steadily increased over 8 h post-infection (Fig. 6D and E). Cells positive for cleaved caspase-3 began appearing at 4 h after T4SS^+^ *Legionella* infection*,* but not during infection with T4SS^+^Δ*7 Legionella* (Fig. 6D and E)*,* indicating that the presence of cleaved caspase-3 depends on effector blockade of host translation. Notably, we did not find individual T4SS^+^ *Legionella*-infected DCs positive for both ASC specks and cleaved caspase-3 (Fig. 6D and E). These data indicate that infected DCs do not simultaneously undergo pyroptosis and apoptosis, but instead exhibit heterogeneity at the single-cell level, even when the two pathways kinetically overlap. Thus, a subset of DCs initially engages rapid pyroptosis, whereas another subset of DCs eventually undergoes apoptosis in response to T4SS effector blockade of host translation.

We next asked whether both pyroptosis and effector-triggered apoptosis restrict *Legionella* replication within DCs. Although *Legionella* replication within *Gsdmd^−/−^* DCs infected with T4SS^+^ *Legionella* was slightly higher than WT DCs infected with T4SS^+^ *Legionella*, it was not statistically significant (Fig. 6F). In contrast, bacterial replication was significantly increased in WT DCs infected with T4SS^+^Δ*7 Legionella* compared to WT DCs infected with T4SS^+^ *Legionella* (Fig. 6F). We observed the greatest increase in bacterial replication within *Gsdmd^−/−^* DCs infected with T4SS^+^ Δ*7 Legionella*, which do not activate pyroptosis nor effector-triggered apoptosis (Fig. 6F). Collectively, these results indicate that effector-triggered apoptosis restricts *Legionella* replication to a greater extent than pyroptosis, but that both are required for maximal restriction of *Legionella* by DCs.

## DISCUSSION

**Fig 7:**
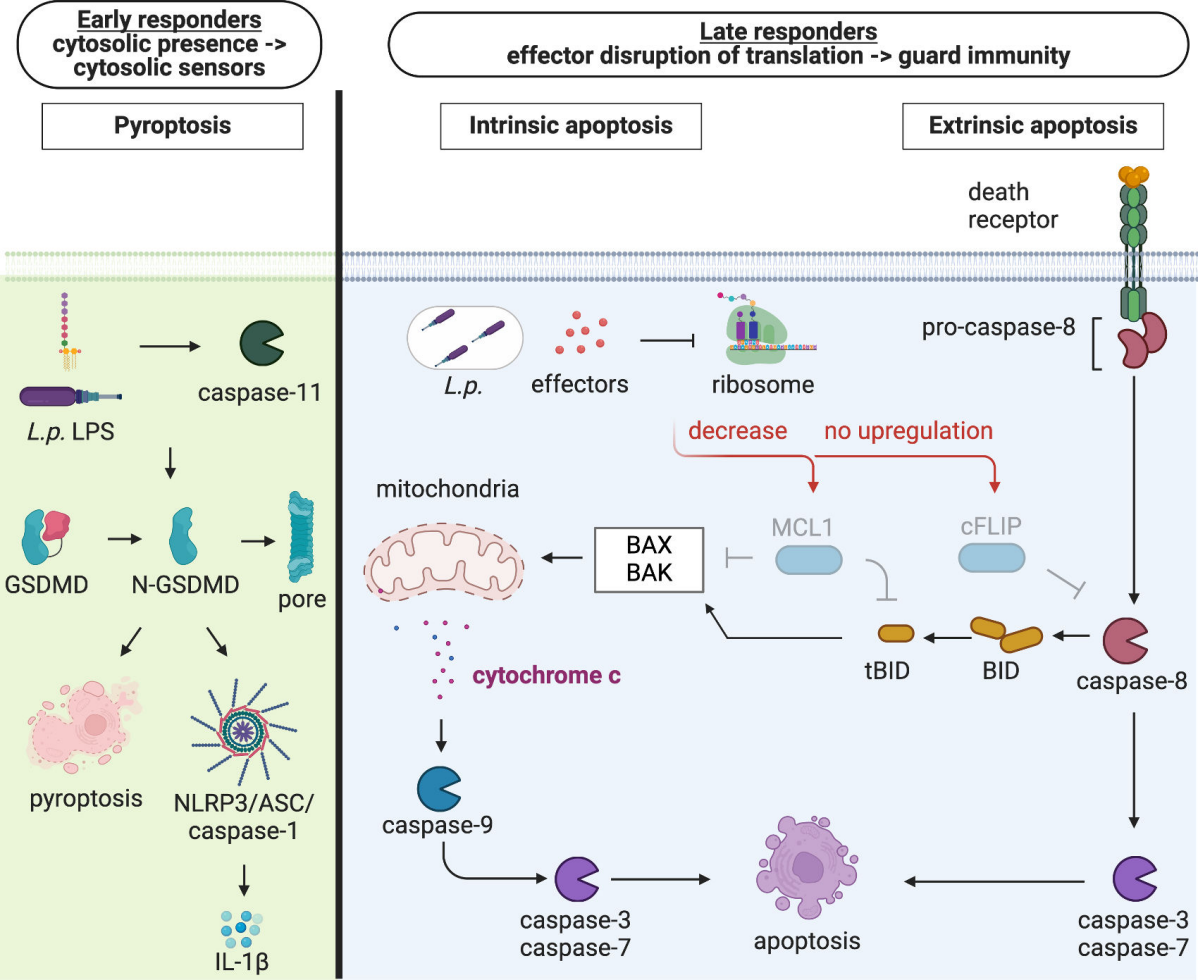
Model of dendritic cell activation of pyroptosis and effector-triggered apoptosis in response to *Legionella* infection. At the early stages of infection (left), some DCs activate caspase-11-dependent pyroptosis in response to *Legionella* LPS in the host cytosol. At the later stages of infection (right), in other DCs, *Legionella* translocates T4SS effectors that block host protein synthesis, which leads to decreased levels of pro-survival molecules that guard host translation, resulting in apoptosis. Figure created with BioRender.

In this study, we employed *Legionella pneumophila* to understand how GM-CSF-derived DCs undergo cell death to restrict intracellular bacterial replication. Our findings show that DCs engage extrinsic and intrinsic apoptosis as an effector-triggered immune response to *Legionella* infection. DC apoptosis was triggered by a subset of *Legionella* T4SS effectors that block host protein synthesis, leading to decreased levels of the pro-survival proteins Mcl-1 and cFLIP. We also found that DCs express lower baseline levels of the pro-survival factors Mcl-1, Bcl-XL, and cFLIP compared to macrophages, which may account for why DCs undergo rapid apoptosis upon bacterial blockade of host translation, whereas macrophages do not. In addition to apoptosis, infected DCs underwent rapid caspase-11 and NLRP3 inflammasome activation. There was considerable heterogeneity at the single-cell level, as individual DCs activated either pyroptosis or apoptosis. Finally, both pyroptosis and effector-triggered apoptosis were required for maximal restriction of *Legionella* replication in DCs (Fig. 7). Thus, DCs employ both effector-triggered apoptosis and pyroptosis to control *Legionella* infection.

The “guard” hypothesis proposes that host proteins monitor cellular processes that are common targets of pathogen effectors, thereby acting as guards of homeostasis (2–4, 107, 108). Host protein synthesis is one such cellular process that our study and others have shown is monitored (18, 57, 93, 109–112). Bcl-2 family proteins and cFLIP guard host translation against chemical or pathogen inhibitors (57, 76, 77, 79–81). Mcl-1 and cFLIP, with half-lives of 30 min and 2 h, respectively, are rapidly depleted upon translational inhibition by cycloheximide, leading to apoptosis (76, 88). In keratinocytes, viral blockade of mRNA translation depletes Mcl-1 and inactivates Bcl-XL, thereby triggering pyroptosis (57). Similarly, NF-κB-dependent cFLIP expression is blocked by inhibitors of NF-κB or mRNA translation, leading to apoptosis (85–88). We found that *Legionella*-induced translation inhibition led to reduced Mcl-1 and cFLIP levels, coinciding with DC apoptosis. Our findings suggest that Mcl-1 and cFLIP act as guards of host protein synthesis and sense bacterial inhibition of host translation in DCs. It will be of interest to uncover the role of these proteins in guarding host translation during infection with other bacterial pathogens in DCs and other cell types.

Our study indicates that GM-CSF-derived DCs possess unique properties that enable them to activate pyroptosis and apoptosis more quickly than macrophages following bacterial infection. While unprimed macrophages require several hours post-*Legionella* infection to activate caspase-11 (36, 37, 40), unprimed DCs rapidly undergo caspase-11-dependent pyroptosis within 1 h post-infection. This rapid response was TNF-dependent but not linked to increased caspase-11 expression, as previously found in TNF-primed macrophages that also undergo rapid caspase-11 activation (40), suggesting that TNF promotes rapid pyroptosis in DCs through other mechanisms. Moreover, infected DCs undergo apoptosis in response to effector blockade of host protein synthesis, unlike macrophages, which do not (18). We show that DCs express lower levels of the pro-survival proteins Bcl-XL, Mcl-1, and cFLIP than macrophages, potentially explaining why DCs are more sensitive than macrophages to apoptosis in response to disrupted protein synthesis. Speir et al. found that while Mcl-1 levels decrease in *Legionella-*infected macrophages, Bcl-XL levels remain high, thus preventing apoptosis and enabling bacterial replication (113). Depleting or inhibiting Bcl-XL induces macrophage apoptosis and restricts *Legionella* replication (113). Together, our work suggests that DCs are uniquely poised for apoptosis upon protein synthesis blockade due to their specific balance of pro- and anti-apoptotic proteins. Future studies should examine whether infected DCs undergo pyroptosis or effector-triggered apoptosis *in vivo*.

It is unclear why DCs and macrophages behave so disparately upon intracellular bacterial infection. As antigen-presenting cells, DCs traffic from the site of infection to the lymph nodes, where they activate adaptive immunity (65–67). If DCs harbor viable pathogens during infection, they may act as a Trojan horse and transport pathogens to other sites, which would be disadvantageous for the host. Perhaps DCs have evolved to rapidly die in response to bacterial pathogens to prevent bacterial dissemination. This finding may extend to other pathogens, as *Salmonella* Typhimurium, *Mycobacterium tuberculosis*, *Listeria monocytogenes*, vaccinia virus, and influenza virus infect but do not replicate within DCs (114–118). In turn, other pathogens may have evolved strategies to replicate within DCs and promote their dissemination.

Previous studies indicate that in other settings, cells undergo PANoptosis, a cell death pathway that involves the simultaneous activation of pyroptosis, apoptosis, and necroptosis (119, 120). Our data indicate that individual *Legionella-*infected DCs undergo either apoptosis or pyroptosis, but not both, indicating that PANoptosis does not occur in our system (Fig. 6D and E). It is unclear what determines whether an individual *Legionella-*infected DC will die by one pathway or another. Perhaps it is due to differential expression of anti-apoptotic factors or inflammasome components at the single-cell level. Alternatively, GM-CSF-derived bone marrow cultures are heterogeneous, as they contain DCs in several differentiation stages as well as monocyte-derived macrophages (121, 122). Thus, depending on their differentiation state, perhaps an individual infected cell dies by either pyroptosis or apoptosis. Alternatively, maybe differences in the rates of effector translocation by individual bacteria trigger apoptosis or pyroptosis in a given infected DC. Additional studies are needed to better understand the mechanisms underlying the heterogeneity of cell death decisions in *Legionella-*infected BMDCs at a single-cell level. It would also be of interest to investigate how different DC subsets interact with *Legionella* during *in vitro* and *in vivo* infection.

One of the most important functions of DCs is to present antigens to lymphocytes to initiate adaptive immune responses (65–67). How DC death influences adaptive immunity during *Legionella* infection remains to be determined. Efferocytosis of dying DCs could provide antigens for uninfected bystander cells to take up and present to T cells, as well as provide signals that direct lymphocyte differentiation. For example, phagocytosis of infected apoptotic cells by uninfected DCs induces a combination of cytokines that promotes T_H_17 differentiation (123). Engulfment of dying cells could also promote cross-priming of CD8^+^ T cells, as has been shown in other settings (124). Future work is needed to understand the consequences of DC death on the instruction of adaptive immunity and infection outcome.

Altogether, this study provides new insight into how DCs detect and restrict the intracellular bacterial pathogen *Legionella*. Early during infection, some DCs activate the caspase-11 and NLRP3 inflammasomes (Fig. 7). As the infection progresses, other DCs undergo effector-triggered apoptosis due to bacterial blockade of host protein synthesis decreasing levels of the pro-survival proteins Mcl-1 and cFLIP (Fig. 7). Our findings lead us to propose a model where Mcl-1 and cFLIP act as guard proteins that respond to bacterial pathogen-mediated disruption of host translation in DCs (Fig. 7). These studies provide a foundation for understanding whether other bacterial pathogens similarly activate pyroptosis and effector-triggered apoptosis in DCs and other cell types.

## MATERIALS AND METHODS

### Mice

All animals were housed and bred in specific-pathogen-free conditions. C57BL/6, *Nlrp3^−/−^* (125), *Casp1^−/−^* (126), and ASC-citrine reporter (127) mice were purchased from Jackson Laboratories. *Tnf^−/−^* (128) mice were bred in-house. *Ripk3^−/−^* (129), *Ripk3^−/−^Casp8^−/−^* (129, 130), *Gsdmd^−/−^* (126), *Gsdmd^−/−^Gsdme^−/−^* (40), and *Casp1^−/−^Casp11^−/−^* (131) bone marrow was provided from mice that were bred in-house by the laboratory of Dr. Igor Brodsky.

### Mouse bone marrow-derived macrophage and dendritic cell culture

Bone marrow was harvested from mice and differentiated into macrophages by culturing on 10 cm non-tissue culture-treated (non-TC) dishes at 37°C in RPMI 1640 containing 30% L929 cell supernatant, 20% fetal bovine serum (FBS), 100 IU/mL penicillin, and 100 µg/mL streptomycin. Cells were fed at day 4 with 10 mL of media. One day prior to infection (day 8), cells were plated in RPMI 1640 containing 15% L929 cell supernatant and 10% FBS. Macrophages were plated at 1 × 10^5^ cells per well in 48-well TC-treated plates and incubated at 37°C.

To generate BMDCs, bone marrow cells were differentiated by culturing on 10 cm non-TC dishes in RPMI 1640 containing 10% FBS, 50 µM 2-mercaptoethanol, 1% L-glutamine, 100 IU/mL penicillin, 100 µg/mL streptomycin, and 20 ng/mL recombinant GM-CSF (PeproTech 315-03). Cells were fed 5 mL of media on days 3 and 5. One day prior to infection (day 8), cells were plated in media containing 10% FBS, 50 µM 2-mercaptoethanol, 1% L-glutamine, and 5 ng/mL GM-CSF. Dendritic cells were plated at 2 × 10^6^ cells per well in 6-well non-TC plates, 2 × 10^5^ cells per well in 24-well non-TC plates, or 1 × 10^5^ cells per well in 48-well non-TC treated plates, followed by incubation at 37°C.

### Bacterial culture

*Legionella pneumophila* strains derived from the Philadelphia 1 Lp02 background (*thyA rpsL hsdR*) (50) were cultured on charcoal yeast extract agar (CYE) containing streptomycin for 4 days. Single colonies were isolated and cultured on CYE plates for 48 h prior to use for infection in experiments. Lp02 DotA- and flagellin-deficient (Δ*dotA*Δ*flaA*) and flagellin-deficient (Δ*flaA*) mutant strains were used (50, 132). The Lp02 Δ*7*Δ*flaA* strain lacks flagellin and seven effectors that block host protein synthesis (∆*lgt1*∆l*gt2*∆*lgt3*∆*sidI*∆*sidL*∆*legK4*∆*ravX*) (55). For experiments assessing bacterial replication, we used Lp02 strains carrying the plasmid pJB908, which carries a WT copy of *thyA* encoding thymidine synthetase (18). pJB908 encoding Lgt2 or a catalytically inactive point mutant (Lgt2*) was transformed into electrocompetent Lp02 Δ*7*Δ*flaA* cells (18) and plated on CYE plates containing chloramphenicol. Transformed colonies were screened via PCR for the plasmid. See Table S1 for a summary of the bacterial strains and plasmids used in this study.

### Bacterial infections

Heavy patches of *L. pneumophila* grown on CYE agar plates for 48 h were resuspended in sterile PBS. Cells were infected at a multiplicity of infection (MOI) of 50, unless otherwise stated. Infected cells were centrifuged and incubated at 37°C. For all experiments, control cells were mock-infected with PBS.

### Intracellular replication assays

Intracellular replication of *L. pneumophila* in BMDMs was measured as described previously (133) and modified slightly for BMDCs (69). DCs were infected at an MOI of 10 with *L. pneumophila*. Plates were centrifuged at 1,200 rpm for 5 min and incubated at 37°C for 30 min. Cells were harvested from wells, and anti-CD11c magnetic beads (Cat# 30-125-835; Miltenyi Biotec) were added. DCs were positively selected using MS columns (Miltenyi Biotec) and the MiniMACS magnetic separator, and extracellular bacteria were removed by washing the column with PBS containing 2% FBS. DCs were eluted from the column, and 2 × 10^5^ cells were plated in non-TC 24-well plates. At indicated times after infection, adherent and non-adherent cells were harvested from wells, lysed with sterile H_2_O, and combined with culture supernatants. Dilutions of the combined cell lysate and supernatant were plated on CYE plates to enumerate bacterial CFUs. Data are the mean CFUs recovered from three independent wells ± standard error of the mean (SEM). The fold increase in intracellular bacterial replication was calculated by determining the fold increase in CFUs at day 1 compared to the internalized CFUs at day 0.

### Cell death assays

Cells were infected as described above and assayed for cell death by measuring lactate dehydrogenase (LDH) activity in the supernatant using an LDH Cytotoxicity Detection Kit (Clontech) and normalized to mock-infected cells as well as cells treated with 1% Triton X-100 to establish maximum LDH release.

To measure cytotoxicity via PI uptake, cells were infected in 96-well black-walled TC plates. At the time of infection, 5 µM PI was added to the plate reader media (20 mM HEPES buffer and 10% FBS in Hank’s balanced salt solution). Cells were then centrifuged at 1,200 rpm for 5 min and allowed to equilibrate to 37°C for 10 min. PI uptake into cells was then measured at an excitation wavelength of 530 nm and an emission wavelength of 617 nm. PI uptake was normalized to mock-infected cells and 1% Triton X-100-treated cells.

### Immunoblot analysis

Infected cells were lysed with 1× SDS-PAGE sample buffer. Protein samples were boiled for 5 min, separated by SDS-PAGE, and transferred to Immobilon-P membranes (Millipore). Samples were then probed with antibodies specific for caspase-3 (Cell Signaling 9662), caspase-7 (Cell Signaling 9492), caspase-8 (Cell Signaling 4790), caspase-9 (Cell Signaling 9508), BID (R&D MAB860), Mcl-1 (Cell Signaling 5453), Bcl-XL (Cell Signaling 2764), cFLIP (Cell Signaling 56343), gasdermin-D (abcam 209845), caspase-11 (Sigma C1354), and caspase-1 (Genentech). As a loading control, all blots were probed with anti-β-actin (Cell Signaling 4967L). Detection was performed with HRP-conjugated anti-mouse IgG (Cell Signaling F00011), anti-rabbit IgG (Cell Signaling 7074S), or anti-rat IgG (Cell Signaling 7077).

Densitometry of pro-apoptotic proteins (Mcl-1, Bcl-XL, and cFLIP) was evaluated using ImageJ, normalizing to the corresponding β-actin band for that condition and time point, and further normalized to mock-infected cells.

### SUnSET assay

To assess global host protein synthesis, we utilized the SUnSET assay (82). Briefly, BMDCs were infected at an MOI of 10 for 4 h. As a positive control, cells were treated with cycloheximide (1 µg/mL) for 4 h. After 4 h of infection or cycloheximide treatment, puromycin was added at 10 µg/mL for 1 h. Lysates were harvested and processed as described above. Blots were probed for puromycin incorporation with an anti-puromycin antibody (DSHB Cat# PMY-2A4, RRID:AB_2619605). Anti-puromycin staining was quantified using ImageJ.

### Enzyme-linked immunosorbent assays

Harvested supernatants from infected cells were assayed using ELISA kits for mouse IL-1α (R&D Systems) and IL-1β (BD Biosciences) following the manufacturer’s instructions.

### Fluorescence and confocal microscopy

BMDCs were seeded at a density of 8 × 10^5^ cells/mL on round, poly-l-ornithine-coated, 12 mm diameter glass coverslips (Electron Microscopy Sciences 72230-01) and allowed to adhere overnight in non-TC 24-well plates. Cells were then infected with *Legionella* strains at an MOI of 50. At the indicated time points, cells were washed three times with PBS and fixed with 4% paraformaldehyde. Cells were stained with primary antibody against rabbit anti-cleaved caspase-3 (Cell Signaling 9664), followed by anti-rabbit AF647 (Invitrogen A21245). Following nuclear counterstain with Hoechst 33342 (1 μg/mL; Thermo Fisher #62249), cells were mounted on glass slides with ProLong Glass (Invitrogen P36882) and dried overnight. Slides were imaged using a Zeiss LSM 980 confocal microscope at a single z-plane per field with lasers optimized for Cy5 (far-red), citrine (yellow), and CellTracker Violet (blue) emission spectra through a ×63 objective. The presence of ASC specks and cleaved caspase-3 was assessed in individual cells. An ASC speck was defined as a distinct, high-fluorescent perinuclear cluster of citrine signal. Each experiment was analyzed for an average of 100–150 cells per condition. Three independent experiments were quantified and graphed.

### Statistical analyses

Graphing and statistical analysis were carried out in GraphPad Prism 10. In comparisons between two groups, unpaired Student’s *t*-test or one-way analysis of variance (ANOVA) followed by Tukey honestly significant difference (HSD) test was utilized to determine significance. In comparisons between more than two groups, two-way ANOVA was utilized to determine significance, followed by Šídák’s post-test or Dunnett’s post-test. Differences were considered significant when the *P* value was <0.05.

## SUPPLEMENTAL MATERIAL

Figure S1 mbio.01257-25-s0001.tiff Caspase-8 is not the sole driver of apoptosis in *Legionella*-infected dendritic cells.

Figure S2 mbio.01257-25-s0002.tiff Effector-triggered apoptosis does not account for all cell death occurring in dendritic cells.

Figure S3 mbio.01257-25-s0003.tiff *Legionella*-mediated block in host protein synthesis decreases IL-1 secretion in dendritic cells.

Figure S4 mbio.01257-25-s0004.tiff TNF promotes pyroptosis but not cell-extrinsic apoptosis in dendritic cells during *Legionella* infection.

Supplemental Legends mbio.01257-25-s0005.docx Legends for supplemental figures and table.

Table S1 mbio.01257-25-s0006.tiff Summary of the bacterial strains and plasmids used in this study.

ASM does not own the copyrights to Supplemental Material that may be linked to, or accessed through, an article. The authors have granted ASM a non-exclusive, world-wide license to publish the Supplemental Material files. Please contact the corresponding author directly for reuse.
