# Supplementary figures and images for: Dendritic cells activate pyroptosis and effector-triggered apoptosis to restrict Legionella infection
Source: mBio. 2025 Jun 18;16(7):e01257-25. doi: 10.1128/mbio.01257-25 (PMC12239575; doi:10.1128/mbio.01257-25)

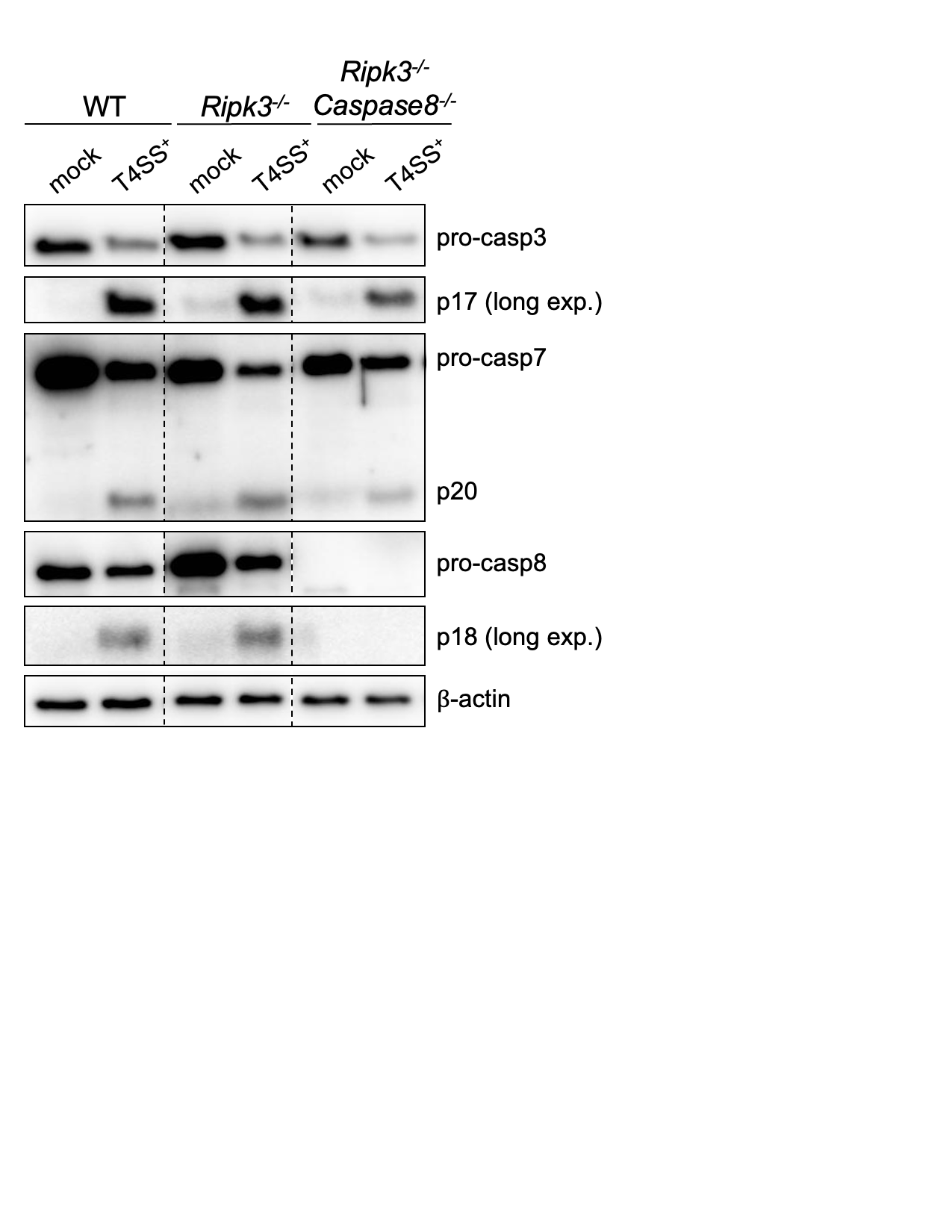

Supplement: Figure S1 — Caspase-8 is not the sole driver of apoptosis in Legionella-infected dendritic cells. [file mbio.01257-25-s0001.tiff]

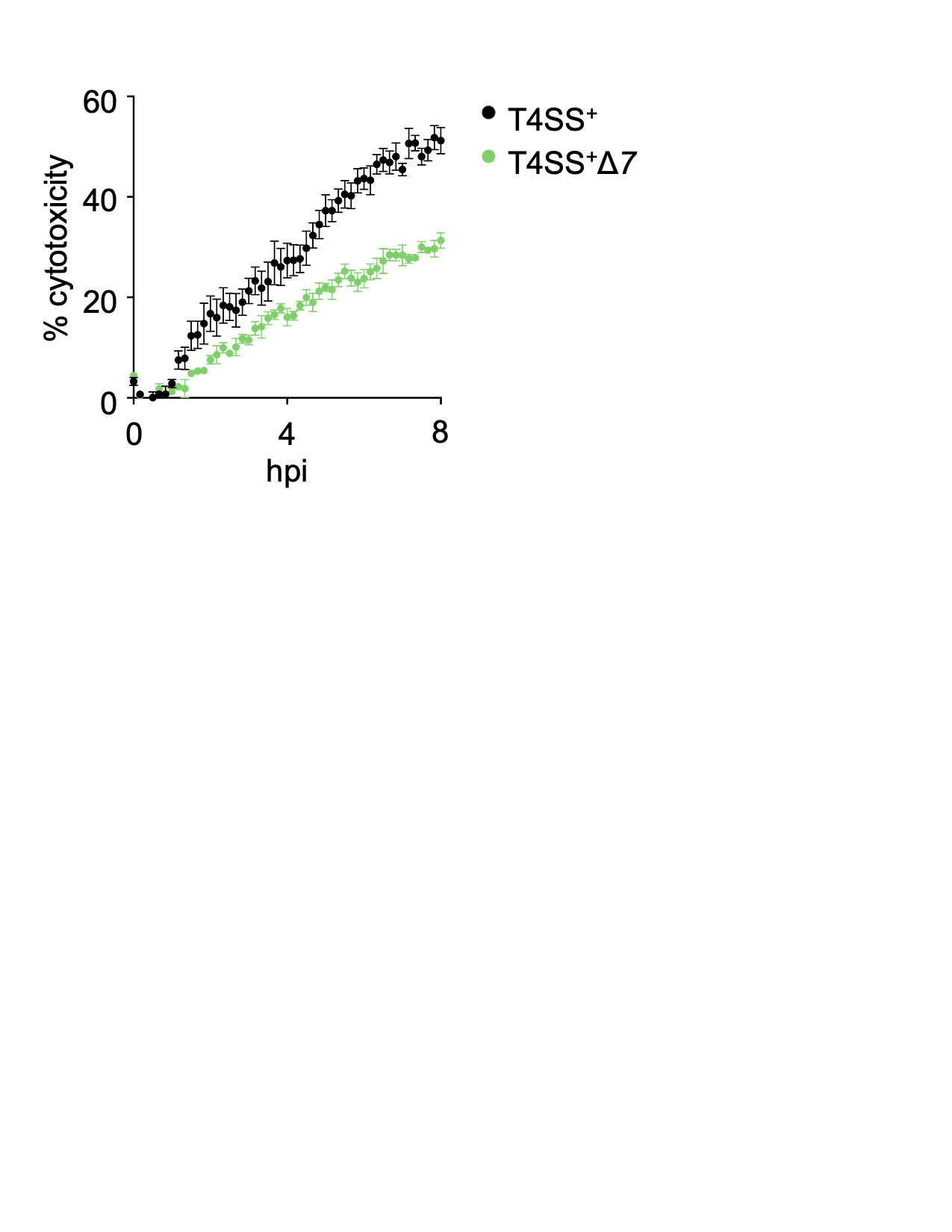

Supplement: Figure S2 — Effector-triggered apoptosis does not account for all cell death occurring in dendritic cells. [file mbio.01257-25-s0002.tiff]

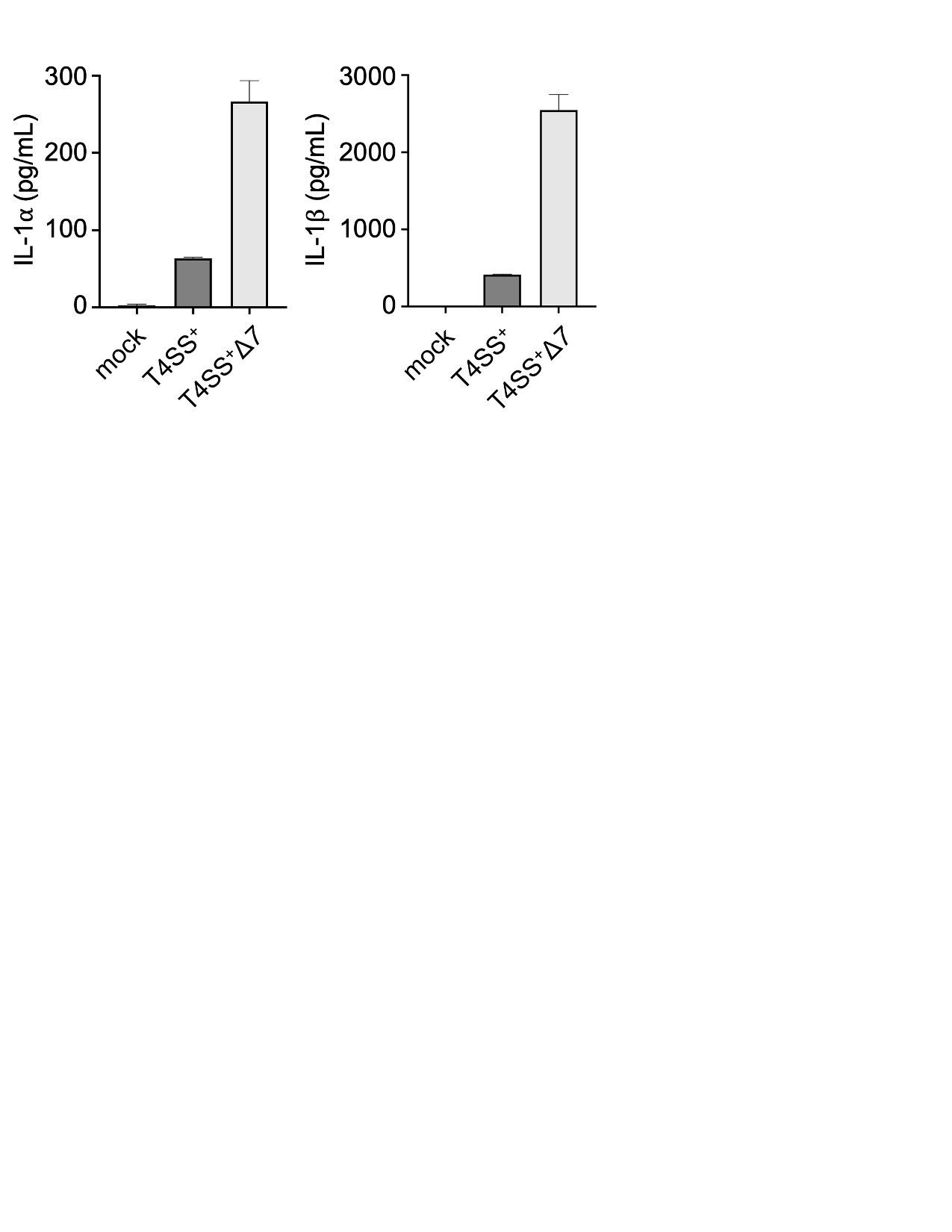

Supplement: Figure S3 — Legionella-mediated block in host protein synthesis decreases IL-1 secretion in dendritic cells. [file mbio.01257-25-s0003.tiff]

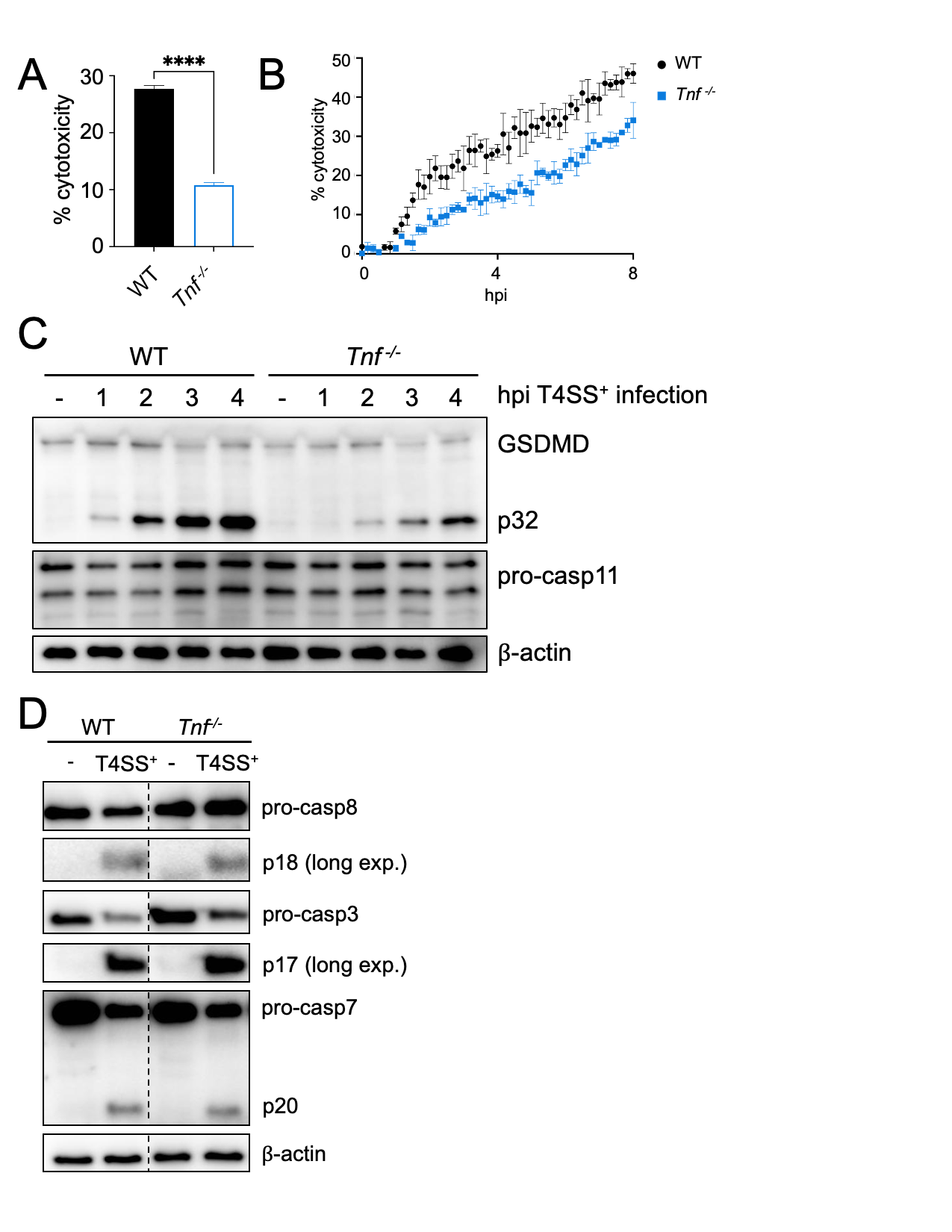

Supplement: Figure S4 — TNF promotes pyroptosis but not cell-extrinsic apoptosis in dendritic cells during Legionella infection. [file mbio.01257-25-s0004.tiff]

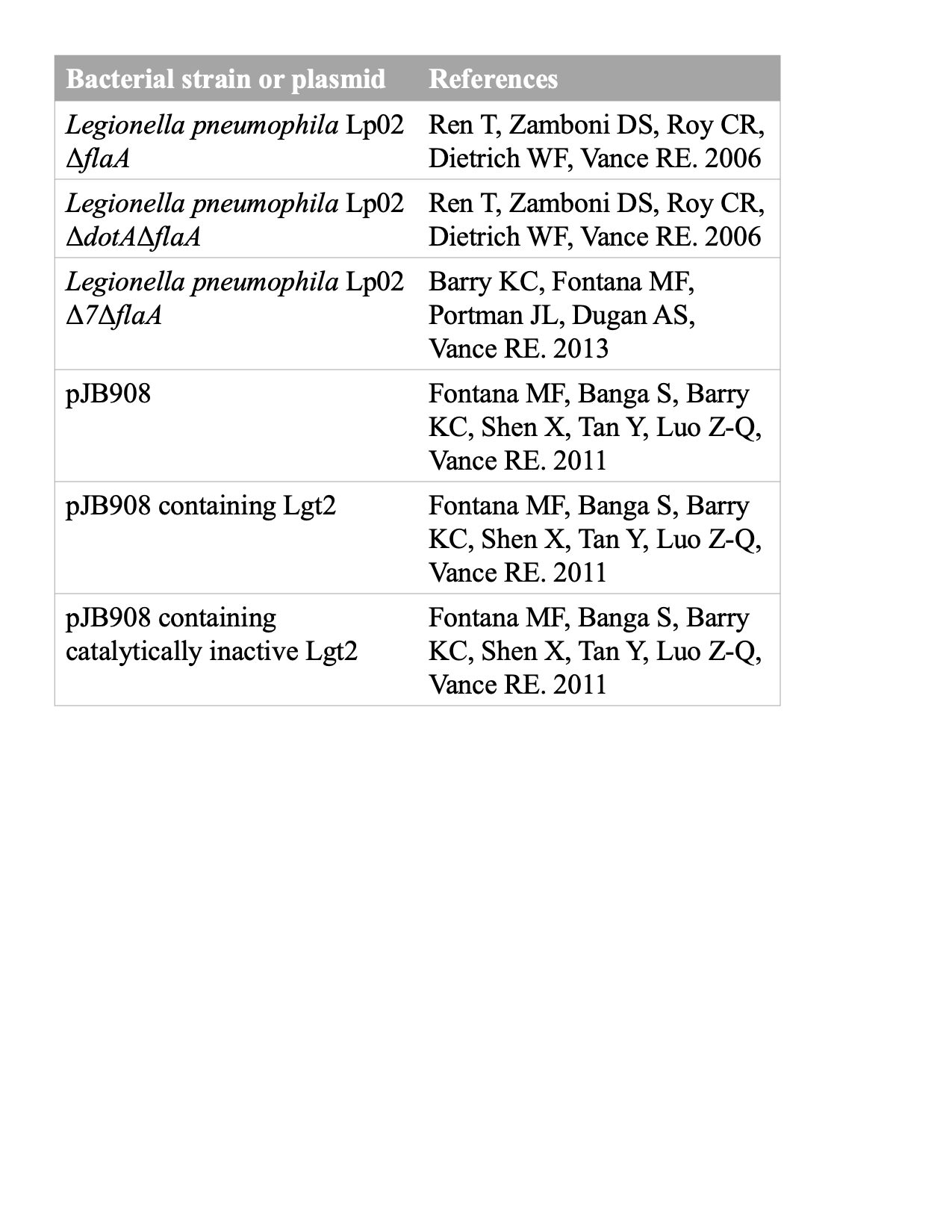

Supplement: Table S1 — Summary of the bacterial strains and plasmids used in this study. [file mbio.01257-25-s0006.tiff]
