# Supplementary material for: Dendritic cells activate pyroptosis and effector-triggered apoptosis to restrict Legionella infection
Source: mBio. 2025 Jun 18;16(7):e01257-25. doi: 10.1128/mbio.01257-25 (PMC12239575; doi:10.1128/mbio.01257-25)
Supplement: Supplemental Legends — Legends for supplemental figures and table. [file mbio.01257-25-s0005.docx]

**Supplemental Figures**

**Figure S1. Caspase-8 is not the sole driver of apoptosis in *Legionella*-infected dendritic cells.** WT, *Ripk3^-/-^*, or *Ripk3^-/-^Casp8^-/-^* BMDCs were mock-infected or infected with T4SS^+^ *Legionella* at a MOI of 50 for 4hrs. Immunoblot analysis was performed on cell lysates for caspase-3, caspase-7, caspase-8, and β-actin as a loading control. The immunoblot was subjected to a longer exposure time to visualize the cleaved forms of caspase-3 and caspase-8. Shown are lanes that were cropped from the same immunoblots, with the dashed line denoting where lanes were cropped. long exp., long exposure. Data shown are representative of at least two independent experiments.

**Figure S2. Effector-triggered apoptosis does not account for all cell death occurring in dendritic cells.** BMDCs were infected with T4SS^+^ or T4SS^+^Δ*7* *Legionella* at an MOI of 50 and cytotoxicity was measured using PI uptake assay. Data shown are representative of at least three independent experiments.

**Figure S3. *Legionella*-mediated block in host protein synthesis decreases IL-1 secretion in dendritic cells.** WT BMDCs were mock-infected or infected with T4SS^+^ or T4SS^+^Δ*7* *Legionella* at an MOI of 50. Cytokine release was measured at 4hrs post-infection by ELISA. Data shown are representative of at least three independent experiments.

**Figure S4.** **TNF promotes pyroptosis but not cell-extrinsic apoptosis in dendritic cells during *Legionella* infection.** (A-B) WT or *Tnf^-/-^* BMDCs were infected with T4SS^+^ *Legionella* at an MOI of 50. Cytotoxicity was measured by PI uptake (A) or LDH release assay at 4hrs post-infection (B). (C) WT or *Tnf^-/-^* BMDCs were mock-infected (represented as “-“) or infected with T4SS^+^ *Legionella* at an MOI of 50 for 1, 2, 3, or 4 hrs. Immunoblot analysis was performed on cell lysates for GSDMD, caspase-11, and β-actin as a loading control. (D) WT or *Tnf^-/-^* BMDCs were mock-infected (represented as “-“) or infected with T4SS^+^ *Legionella* at an MOI of 50 for 4 hrs. Immunoblot analysis was performed on cell lysates for caspase-8, caspase-3, caspase-7, and β-actin as a loading control. The immunoblot was subjected to a longer exposure time to visualize the cleaved forms of caspase-8 and caspase-3. Shown are lanes that were cropped from the same immunoblots, with the dashed line denoting where lanes were cropped. long exp., long exposure. hpi, hours post-infection. Data shown are representative of at least two (C,D) or three (A,B) independent experiments. Graphs show the mean ± SEM of triplicate wells. Data were analyzed by unpaired Student’s t-test; ****, P < 0.0001.

**Table S1**. **Summary of the bacterial strains and plasmids used in this study.**
